# Sarcomatoid Dedifferentiation as a Predictor of Cancer-Specific Mortality in Surgically Treated Localized Renal Cell Carcinoma

**DOI:** 10.1245/s10434-024-15431-5

**Published:** 2024-05-21

**Authors:** Reha-Baris Incesu, Simone Morra, Lukas Scheipner, Andrea Baudo, Cristina Cano Garcia, Francesco Barletta, Anis Assad, Zhe Tian, Fred Saad, Shahrokh F. Shariat, Alberto Briganti, Felix K. H. Chun, Luca Carmignani, Sascha Ahyai, Nicola Longo, Derya Tilki, Markus Graefen, Pierre I. Karakiewicz

**Affiliations:** 1 Cancer Prognostics and Health Outcomes Unit, Division of Urology, University of Montréal Health Center, Montréal, QC Canada; 2 Martini-Klinik Prostate Cancer Center, University Hospital Hamburg-Eppendorf, Hamburg, Germany; 3 Department of Neurosciences, Reproductive Sciences and Odontostomatology, University of Naples Federico II, Naples, Italy; 4 Department of Urology, Medical University of Graz, Graz, Austria; 5 Department of Urology, IRCCS Ospedale Galeazzi – Sant’Ambrogio, Milan, Italy; 6 Department of Urology, IRCCS Policlinico San Donato, Milan, Italy; 7 Department of Urology, University Hospital Frankfurt, Goethe University Frankfurt am Main, Frankfurt am Main, Germany; 8 Gianfranco Soldera Prostate Cancer Lab, Unit of Urology/Division of Oncology, IRCCS San Raffaele Scientific Institute, Milan, Italy; 9 Vita-Salute San Raffaele University, Milan, Italy; 10 Department of Urology, Comprehensive Cancer Center, Medical University of Vienna, Vienna, Austria; 11 Department of Urology, Weill Cornell Medical College, New York, New York USA; 12 Department of Urology, University of Texas Southwestern Medical Center, Dallas, TX USA; 13 Hourani Center of Applied Scientific Research, Al-Ahliyya Amman University, Amman, Jordan; 14 Department of Urology, University Hospital Hamburg-Eppendorf, Hamburg, Germany; 15 Department of Urology, Koc University Hospital, Istanbul, Turkey

**Keywords:** Sarcomatoid dedifferentiation, Renal cell carcinoma, Tumor grade, Cancer-specific mortality

## Abstract

**Background:**

In contemporary surgically treated patients with localized high-grade (G3 or G4) clear-cell renal cell carcinoma (ccRCC), it is not known whether presence of sarcomatoid dedifferentiation is an independent predictor and/or an effect modifier, when cancer-specific mortality (CSM) represents an endpoint.

**Methods:**

Within the Surveillance, Epidemiology, and End Results database, all surgically treated localized high-grade ccRCC patients treated between 2010 and 2020 were identified. Univariable and multivariable Cox-regression models were used.

**Results:**

In 18,853 surgically treated localized high-grade (G3 or G4) ccRCC patients, 5-year CSM-free survival was 87% (62% vs. 88% with vs. without sarcomatoid dedifferentiation, *p *< 0.001). Presence of sarcomatoid dedifferentiation was an independent predictor of higher CSM (hazard ratio [HR] 1.8, *p *< 0.001). In univariable survival analyses predicting CSM, presence versus absence of sarcomatoid dedifferentiation in G3 versus G4 yielded the following hazard ratios: HR 1.0 in absent sarcomatoid dedifferentiation in G3; HR 2.7 (*p *< 0.001) in absent sarcomatoid dedifferentiation in G4; HR 3.9 (*p *< 0.001) in present sarcomatoid dedifferentiation in G3; HR 5.1 (*p *< 0.001) in present sarcomatoid dedifferentiation in G4. Finally, in multivariable Cox-regression analyses, the interaction terms defining present versus absent sarcomatoid dedifferentiation in G3 versus G4 represented independent predictors of higher CSM.

**Conclusions:**

In contemporary surgically treated patients with localized high-grade ccRCC, sarcomatoid dedifferentiation is not only an independent multivariable predictor of higher CSM, but also interacts with tumor grade and results in even better ability to predict CSM.

Presence of sarcomatoid dedifferentiation in renal cell carcinoma (RCC) patients represents an established adverse prognostic feature. However, first, it is unknown whether presence of sarcomatoid dedifferentiation represents an independent predictor of higher cancer-specific mortality (CSM) in contemporary surgically treated localized high-grade (G3 or G4) clear-cell renal cell carcinoma (ccRCC) patients. Second, it is unknown what is the magnitude that sarcomatoid dedifferentiation exerts on CSM. Third, it also is unknown whether in contemporary localized high-grade (G3 or G4) ccRCC patients, presence of sarcomatoid dedifferentiation may interact with tumor grade and whether this interaction term may achieve independent predictor status in CSM-analyses. We addressed these three knowledge gaps and formally tested them within the current study. We relied on the Surveillance, Epidemiology and End Results (SEER) database 2010–2020.

## Materials and Methods

### Patients

The current SEER database samples about 34.6% of the United States population and approximates it in demographic composition and cancer incidence. Within the SEER database (2010–2020), we identified patients ≥ 18 years old with histologically confirmed high-grade (G3 or G4) ccRCC (International Classification of Disease for Oncology [ICD-O] site codes C64.9). Only patients treated with partial or radical nephrectomy were included. We initially included all surgically treated patients regardless of tumor grade. Subsequently, we exclusively focused on G3 and G4, because presence of sarcomatoid dedifferentiation represents an extremely rare event in G1 and G2 patients.^1,2^ Moreover, all autopsy or death certificate cases were excluded. Further exclusion criteria consisted of unknown T stage, unknown tumor size, missing follow-up or missing vital status. Moreover, patients with metastatic disease or patients who underwent metastasectomy were excluded.

### Statistical Analyses

Analyses focused on the effect of sarcomatoid dedifferentiation on CSM-free survival in Kaplan-Meier analyses. Subsequently, independent predictor status of sarcomatoid dedifferentiation was tested, alongside all other established clinical and pathological predictors. Subsequently, Cox-regression models were refitted with sarcomatoid dedifferentiation (presence vs. absence) that was first combined with G3 and then combined with G4, according to recorded tumor grade. Finally, the interaction terms defining present versus absent sarcomatoid dedifferentiation in G3 as well as present versus absent sarcomatoid dedifferentiation in G4 were first tested in univariable Cox-regression models. Subsequently, multivariable Cox-regression models were refitted with the interaction terms after inclusion of all standard clinical and pathological predictors (age at diagnosis, T stage, and N stage) (Table 1).

**Table 1:** Univariable and multivariable Cox-regression analyses addressing CSM in 18,853 surgically treated localized high-grade (G3 or G4) clear-cell renal cell carcinoma patients

	Univariable			Multivariable		
	HR	95% CI	*p*	HR	95% CI	*p*
G3 (ref)	1.0	–	*Ref*	1.0	–	*Ref*
G4	3.1	2.8–3.4	**< 0.001**	1.9	1.7–2.1	**< 0.001**
Absence of sarcomatoid (ref)	1.0	–	*Ref*	1.0	–	*Ref*
Presence of sarcomatoid	3.9	3.5–4.4	**< 0.001**	1.8	1.6–2.1	**< 0.001**

Bold values indicate statistically significant

Multivariable adjustment was made for presence versus absence of sarcomatoid dedifferentiation, tumor grade G3 versus G4, age at diagnosis, T stage, and N stage

*HR* hazard ratio; *CI* confidence interval; *CSM* cancer-specific mortality

In all statistical analyses, R software environment for statistical computing and graphics (The R Foundation for Statistical Computing, Vienna Austria, R version 4.2.1) was used.^3^ All tests were two-sided, with a significance level set at *p *< 0.05. Because of the anonymously coded design of the SEER database, study-specific ethics approval was waived by the institutional review board.^4^

## Results

### Descriptive Characteristics

Overall, 18,853 surgically treated localized high-grade (G3 or G4) ccRCC patients were identified (Table 2). Of those, 13,187 (70%) were male versus 5666 (30%) were female. The median age at diagnosis was 63 years (interquartile range [IQR] 55–70). The median tumor size was 55 mm (IQR 36–80). Of all patients, 15,248 (81%) harbored tumor grade G3 versus 3605 (19%) harbored tumor grade G4. Of all patients, 11,644 (62%) harbored T1/T2 stage versus 7209 (38%) harbored T3/T4. Of all patients, 16,595 (88%) harbored N0 stage versus 1940 (10%) N1 versus 318 (2%) Nx.

Sarcomatoid dedifferentiation was present in 1079 (6%) of all patients. According to presence versus absence of sarcomatoid dedifferentiation, the rate of T3/T4 stage was 66% versus 37% (*p *< 0.001). According to presence versus absence of sarcomatoid dedifferentiation, the median tumor size was 80 versus 54 mm (*p *< 0.001). According to presence versus absence of sarcomatoid dedifferentiation, the rate of N1-stage was 21% versus 10% (*p *< 0.001). Of 15,248 G3 patients, 168 (1%) harbored sarcomatoid dedifferentiation versus 911 (25%) of 3605 G4 patients (*p *< 0.001). It is of note that in G1 and G2 patients that did not represent the focus of the current analyses, the rate of sarcomatoid dedifferentiation was 16 (0.3%) of 6195 in G1 versus 88 (0.3%) of 31,138 in G2. According to presence versus absence of sarcomatoid dedifferentiation, the median patient age was 64 versus 63 (*p *= 0.001). According to presence versus absence of sarcomatoid dedifferentiation, the distribution of male patients was 69% versus 70% (*p *= 0.6; Table 2).

**Table 2:** Baseline characteristics of 18,853 surgically treated localized high-grade (G3 or G4) clear-cell renal cell carcinoma patients, within the SEER database (2010–2020), stratified according to presence versus absence of sarcomatoid dedifferentiation

	Overall, *N* = 18,853	Absence of sarcomatoid dedifferentiation *N* = 17,774 (94%)	Presence of sarcomatoid dedifferentiation, *N* = 1079 (6%)	*p*
Median age at diagnosis (IQR)	63 (55, 70)	63 (55, 70)	64 (56, 71)	**0.001**
Median tumor size in mm (IQR)	55 (36, 80)	54 (35, 78)	80 (57, 105)	**< 0.001**
Sex				1.0
Male	13,187 (70%)	12,441 (70%)	746 (69%)	
Female	5666 (30%)	5333 (30%)	333 (31%)	
Tumor grade				**< 0.001**
G3	15,248 (81%)	15,080 (85%)	168 (16%)	
G4	3605 (19%)	2694 (15%)	911 (84%)	
T stage				**< 0.001**
T1/T2	11,644 (62%)	11,274 (63%)	370 (34%)	
T3/T4	7209 (38%)	6500 (37%)	709 (66%)	
N stage				**< 0.001**
N0	16,595 (88%)	15,766 (89%)	829 (77%)	
N1	1940 (10%)	1717 (9.7%)	223 (21%)	
Nx	318 (1.7%)	291 (1.6%)	27 (2.5%)	
Systemic therapy	570 (3.0%)	478 (2.7%)	92 (8.5%)	**< 0.001**

Bold values indicate statistically significant

Data are median (IQR) or *n* (%) unless otherwise indicated

Wilcoxon rank-sum test; Pearson’s chi-square test

### Cancer-specific Mortality of Surgically Treated Localized High-Grade (G3 or G4) ccRCC Patients

**Fig. 1:**
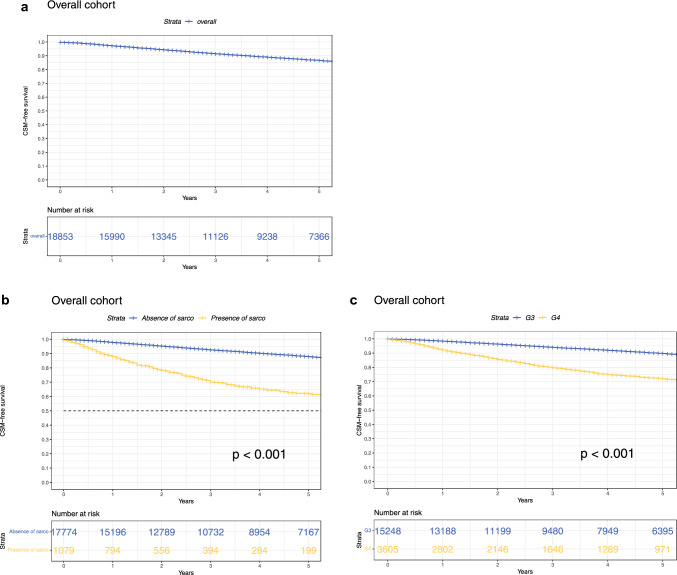
Kaplan–Meier plots illustrating CSM in the overall cohort as well as separately stratified according to presence versus absence of sarcomatoid dedifferentiation and tumor grade G3 versus G4. *CSM* cancer-specific mortality; *Sarco* sarcomatoid dedifferentiation

In 18,853 surgically treated localized high-grade (G3 or G4) ccRCC patients, overall 5-year CSM-free survival was 87% (Fig. 1a). Median CSM-free survival was not reached. After stratification according to presence versus absence of sarcomatoid dedifferentiation (Fig. 1b), 5-year CSM-free survival rates were 62% versus 88%, respectively (*p *< 0.001). After stratification according to tumor grade G4 versus G3 (Fig. 1c), 5-year CSM-free survival rates were 72% versus 90%, respectively (*p *< 0.001). In univariable Cox-regression analyses (Table 1), presence of sarcomatoid dedifferentiation was associated with higher CSM (HR 3.9, 95% confidence interval [CI] 3.5–4.4, *p *< 0.001), compared with absence of sarcomatoid dedifferentiation. In multivariable Cox-regression analyses (Table 1), presence of sarcomatoid dedifferentiation (HR 1.8, 95% CI 1.6–2.1, *p *< 0.001) was independently associated with higher CSM, compared with absence of sarcomatoid dedifferentiation.

**Fig. 2:**
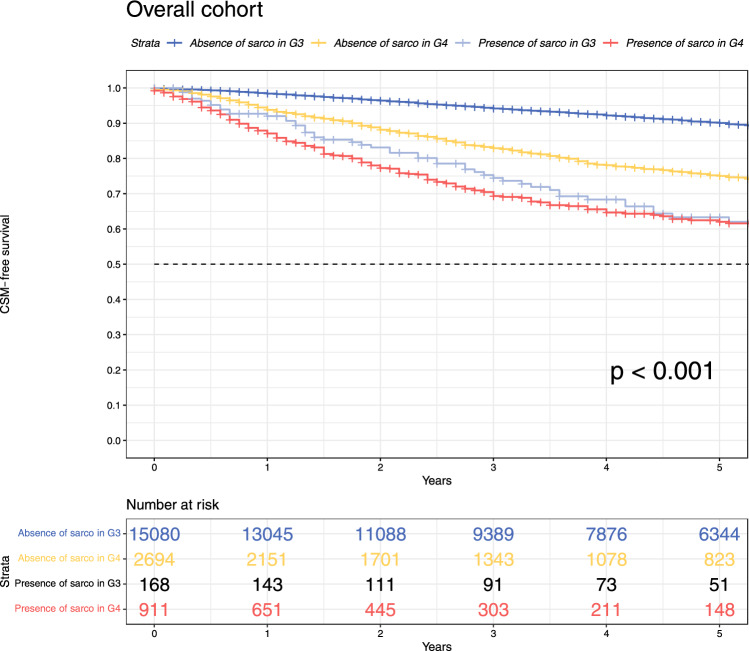
Kaplan–Meier plots illustrating CSM in the overall cohort as well as separately stratified according to four levels of interaction terms: absence of sarcomatoid dedifferentiation in G3 versus absence of sarcomatoid dedifferentiation in G4 versus presence of sarcomatoid dedifferentiation in G3 versus presence of sarcomatoid dedifferentiation in G4. *CSM* cancer-specific mortality; *Sarco* sarcomatoid dedifferentiation

In univariable CSM-models that were refitted with the interaction terms defining present versus absent sarcomatoid dedifferentiation in G3 as well as present versus absent sarcomatoid dedifferentiation in G4 (Fig. 2), the following 5-year CSM-free survival rates were recorded: 90% in absent sarcomatoid dedifferentiation in G3; 75% in absent sarcomatoid dedifferentiation in G4; 63% in present sarcomatoid dedifferentiation in G3; 62% in present sarcomatoid dedifferentiation in G4 (*p *< 0.001). In Cox-regression analyses (Table 3), the associated univariable hazard ratios were as follows: HR 1.0 for absent sarcomatoid dedifferentiation in G3 versus HR 2.7 (95% CI 2.5–3.0, *p *< 0.001) for absent sarcomatoid dedifferentiation in G4 versus HR 3.9 (95% CI 2.9–5.1, *p *< 0.001) for present sarcomatoid dedifferentiation in G3 versus HR 5.1 (95% CI 4.4–5.8, *p *< 0.001) for present sarcomatoid dedifferentiation in G4. After further adjustment for all other clinical and pathological variables (age, T stage, N stage), all variables defining the interaction between sarcomatoid dedifferentiation and G3 as well as sarcomatoid dedifferentiation and G4 (absent sarcomatoid dedifferentiation in G3 vs. absent sarcomatoid dedifferentiation in G4 vs. present sarcomatoid dedifferentiation in G3 vs. present sarcomatoid dedifferentiation in G4) reached independent predictor status (all *p *< 0.001; Table 3).

**Table 3:** Univariable and multivariable Cox-regression analyses addressing CSM in 18,853 surgically treated localized high-grade (G3 or G4) clear-cell renal cell carcinoma patients, according to four levels of interaction terms: absence of sarcomatoid dedifferentiation in G3 versus absence of sarcomatoid dedifferentiation in G4 versus presence of sarcomatoid dedifferentiation in G3 versus presence of sarcomatoid dedifferentiation in G4

	Univariable			Multivariable		
	HR	95% CI	*p*	HR	95% CI	*p*
Absence of sarcomatoid dedifferentiation in G3 (ref)	1.0	–	*Ref*	1.0	–	*Ref*
Absence of sarcomatoid dedifferentiation in G4	2.7	2.5–3.0	**< 0.001**	2.0	1.8–2.3	**< 0.001**
Presence of sarcomatoid dedifferentiation in G3	3.9	2.9–5.1	**< 0.001**	3.1	2.4–4.1	**< 0.001**
Presence of sarcomatoid dedifferentiation in G4	5.1	4.4–5.8	**< 0.001**	3.3	2.9–3.8	**< 0.001**

Bold values indicate statistically significant

Multivariable adjustment was made for age at diagnosis, T stage, and N stage

*HR* hazard ratio; *CI* confidence interval; *CSM* cancer-specific mortality

## Discussion

We performed detailed analyses addressing the ability of sarcomatoid dedifferentiation to predict CSM in isolation and/or in combination with G3 versus G4 in contemporary surgically treated localized high-grade (G3 or G4) ccRCC patients. The current study led to several noteworthy observations.

First, in 18,853 patients treated surgically for localized high-grade (G3 or G4) ccRCC between the years 2010 and 2020, the presence of sarcomatoid dedifferentiation was identified in 1079 (6%) patients. Previous studies relied on mixed populations that included metastatic RCC patients and/or on populations that included surgically treated and nonsurgically treated patients and/or on populations that addressed presence of sarcomatoid dedifferentiation in histological subtypes other than clear-cell.^1,5–8^ To the best of our knowledge, the current analysis represents a first formal, structured, systematic assessment of the effect of sarcomatoid dedifferentiation in a very large contemporary homogeneous group of patients with localized high-grade ccRCC that all underwent nephrectomy. For example, in a previous study, Brookman-May et al. relied on a multi-institutional database (*n* = 8126; 1992–2010) of surgically treated RCC patients.^6^ However, as many as 21% of patients harbored metastatic RCC and an even larger proportion (27%) harbored nonclear-cell histological subtypes.^6^ Similarly, Tully et al. (*n* = 8582; 2010–2015) relied on the National Cancer Database (NCDB) and also included metastatic patients (48%) as well as nonclear cell histological subtypes (48%).^8^ In consequence, the studies of Brookman-May et al. and Tully et al. could not test the effect of sarcomatoid dedifferentiation in surgically treated nonmetastatic RCC of exclusive clear-cell histological subtype, as was done in the current study. Additionally, the study of Tully et al. also is limited by the inability of CSM as an endpoint.^8^ Instead, it only relied on overall mortality (OM), because CSM is not available within the NCDB. This data limitation represents a fatal flaw in analyses focusing on patients with nonmetastatic RCC. An additional limitation of Tully et al. consists of a cohort, where sarcomatoid dedifferentiation was recorded in all patients.^8^ The same methodological limitation also applies to Alevizakos et al.^7^ Lack of inclusion of control patients without sarcomatoid dedifferentiation limits the possibility for comparisons between these two key groups. In consequence, such studies could not test the hypotheses addressed in the current study. Last but not least, of all previous studies that addressed the effect of sarcomatoid dedifferentiation in RCC, only Brookman-May et al.^6^ focused on patients with tumor grade G3 and G4, as was done in the current study. The rationale for excluding G1 and G2, as was done by Brookman-May et al. as well as in the current study, consists of extremely low prevalence of sarcomatoid dedifferentiation in such patients. Indeed, within the current SEER cohort, the prevalence of sarcomatoid dedifferentiation in G1 and G2 was as low as 0.3% and 0.3%. Inclusion of G1 and G2 patients, where sarcomatoid dedifferentiation is virtually invariably absent, dilutes the study population with observations that have no impact on its endpoint. Moreover, the inclusion of such observations may adversely affect the statistical metrics by consuming degrees of freedom and reducing the power of meaningful comparisons. Taken together, the above examples of previous studies addressing the effect of sarcomatoid dedifferentiation in RCC emphasize their important limitations and validate the need for the current study with a strict focus on surgically treated localized high-grade (G3 or G4) ccRCC patients.

Second, patients with present sarcomatoid dedifferentiation exhibited consistently more adverse baseline characteristics, compared with patients with absent sarcomatoid dedifferentiation. Specifically, patients with present sarcomatoid dedifferentiation were older (median age 64 vs. 63, *p *= 0.001), exhibited larger primary tumor size (80 vs. 54 mm, *p *< 0.001), a higher rate of G4 (84 vs. 15%, *p *< 0.001), higher rate of T3/T4 stage (66 vs. 37%, *p *< 0.001), as well as higher rate of N1 stage (21 vs. 10%, *p *< 0.001). These observations indicate higher prevalence of sarcomatoid dedifferentiation according to more aggressive features, such as larger tumor size, higher tumor grade, and higher stage. This observation indirectly validates that sarcomatoid dedifferentiation is correctly considered as a marker of more aggressive natural history in contemporary surgically treated ccRCC patients. To the best of our knowledge, we are the first to validate this notion in a contemporary population of surgically treated localized ccRCC patients.

Third, we focused on the effect of sarcomatoid dedifferentiation on CSM-free survival in the current cohort. Presence of sarcomatoid dedifferentiation discriminated between worse versus better CSM-free survival, when it was analyzed in univariable survival analyses (5-year CSM-free survival 62 vs. 88%, *p *< 0.001). Its independent predictor status was then confirmed in multivariable Cox-regression analyses (HR 1.8, 95% CI 1.6–2.1, *p *< 0.001). Most importantly, the interaction terms defining present versus absent sarcomatoid dedifferentiation in G3, as well as present versus absent sarcomatoid dedifferentiation in G4 that resulted in a four-level variable, exhibited a novel and previously unknown relationship with CSM-free survival. Specifically, 5-year CSM-free survival decreased from the highest (90%) in patients with absent sarcomatoid dedifferentiation in G3 tumors to 75% in patients with absent sarcomatoid dedifferentiation in G4 to 63% in patients with present sarcomatoid dedifferentiation in G3 to 62% in patients with present sarcomatoid dedifferentiation in G4 (*p *< 0.001). These CSM-free rate differences yielded equally impressive differences in hazard ratios that were as follows: HR 1.0, HR 2.7, HR 3.9, HR 5.1. These hazard ratios exhibited a multiplicative effect between present versus absent sarcomatoid dedifferentiation in G3 and present versus absent sarcomatoid dedifferentiation in G4 rather than a simple additive effect. Such increase in hazard rates therefore may be considered as presence of a dose-response effect between these two variables. Finally, in multivariable Cox-regression models (adjusted for all available covariables) that were fitted with the four-level variable defining present versus absent sarcomatoid dedifferentiation in G3 as well as present versus absent sarcomatoid dedifferentiation in G4 (absent sarcomatoid dedifferentiation in G3 vs. absent sarcomatoid dedifferentiation in G4 vs. present sarcomatoid dedifferentiation in G3 vs. present sarcomatoid dedifferentiation in G4), all four levels achieved independent predictor status (all *p *< 0.001). These observations cannot be directly compared with other studies, because no previous study addressed the same endpoints in a similar fashion to our study.

Finally, it is of note that ccRCC patients with presence of sarcomatoid dedifferentiation should invariably be diagnosed as G4 patients according to contemporary recommendations by the World Health Organization (WHO) and the International Society of Urological Pathology (ISUP).^9–11^ However, despite this definition, sarcomatoid dedifferentiation can still occur in tumor tissue with underlying tumor grades other than G4, especially G3. The current study benefits from the large SEER database, where these characteristics (tumor grade, presence or absence of sarcomatoid dedifferentiation) are collected separately without adaption according to the WHO/ISUP recommendations. In consequence, the current study offers a unique opportunity to explore how the interaction between presence of sarcomatoid dedifferentiation and the underlying tumor grade (G3 or G4) affects CSM in contemporary surgically treated localized high-grade (G3 or G4) ccRCC patients.

Taken together, our observations indicate that in contemporary surgically treated localized high-grade (G3 or G4) ccRCC patients, presence or absence of sarcomatoid dedifferentiation resulted in significant CSM rate differences. Additionally, the current study indicates a dose-response effect between present versus absent sarcomatoid dedifferentiation in G3, as well as present versus absent sarcomatoid dedifferentiation in G4 on CSM. The survival detriment associated with presence of sarcomatoid dedifferentiation is dependent of the underlying tumor grade in localized high-grade ccRCC patients. Specifically, the effect of presence of sarcomatoid dedifferentiation is stronger in G4 than the effect of presence of sarcomatoid dedifferentiation in G3. This interaction between sarcomatoid dedifferentiation and tumor grade not only provided better discrimination of CSM rates, but also achieved independent predictor status after adjustment for all standard clinical and pathological variables. The limitation of the importance of sarcomatoid dedifferentiation in localized surgically treated high-grade (G3 or G4) ccRCC consists of its relative rarity (6%).

Despite its novelty, the current study has several limitations. First, all variables were recorded retrospectively. Their quality is not equal to that of a prospective design, where presence of sarcomatoid dedifferentiation is prospectively quantified in all surgical specimen. However, such design is not possible, because sarcomatoid dedifferentiation is too rare in G3/G4 (6%) and virtually absent in G1/G2. In consequence, the limitations of a retrospective design with inherent collection of sarcomatoid dedifferentiation data are the only available option. Second, additional limitations related to lack of central pathology also need to be considered. Third, the lack of percentage of sarcomatoid dedifferentiation is another limitation, since it has been shown that the percentage of sarcomatoid dedifferentiation was independently associated with higher CSM in localized RCC patients.^4^ Fourth, other candidate variables that are not routinely considered should ideally be included in the analyses, such as presence of tumor necrosis, infiltrative growth patterns and/or lymphatic invasion.^12^ Finally, absence of recurrence data might represent a limitation. Ideally, recurrence data would be analyzed along with CSM data. Using recurrence as an endpoint, higher proportions of events could be reported, especially in the very small subgroups of patients with sarcomatoid dedifferentiation. However, the current method that relies on CSM represents a more robust endpoint, since not all patients with a recurrence invariably die of ccRCC. An additional advantage of using CSM as an endpoint instead of OM consists of inherent adjustment for other-cause mortality (OCM). Use of OM as an endpoint is limited by confounding by OCM. Especially in localized ccRCC, the confounding effect of OCM is usually rate-limiting and the analyses that relied on OM are of limited if of any value. Such limitation applies to the NCDB, where CSM is not available. Despite residual limitations, the current study provides a novel perspective on the potential detriments associated with presence of sarcomatoid dedifferentiation in surgically treated localized high-grade ccRCC patients.

## Conclusions

In contemporary surgically treated patients with localized high-grade ccRCC, sarcomatoid dedifferentiation is not only an independent multivariable predictor of higher CSM, but also interacts with tumor grade and results in even better ability to predict CSM.

## Data Availability

All data generated or analyzed during this study are included in this article. Further enquiries can be directed to the corresponding author.
